# Supplementary material for: The Systems Biology Research Tool: evolvable open-source software
Source: BMC Syst Biol. 2008 Jun 29;2:55. doi: 10.1186/1752-0509-2-55 (PMC2446383; doi:10.1186/1752-0509-2-55)
Supplement: Additional file 1 — SBRT Archive. An archive of the current version of the Systems Biology Research Tool. [file 1752-0509-2-55-S1.zip › sbrt-1.4.0/doc/developers_guide/api/sbrt/shell/text/chem/package-use.html]

Uses of Package sbrt.shell.text.chem


|  |  |  |
| --- | --- | --- |
| **Overview** \| **Package** \| Class \| **Use** \| **Tree** \| **Deprecated** \| **Index** \| **Help** | |  |
| PREV   NEXT | **FRAMES**    **NO FRAMES**     **All Classes** |  |


---


## **Uses of Package sbrt.shell.text.chem**

| Packages that use sbrt.shell.text.chem | |
| --- | --- |
| **sbrt.shell.proc.fba** | Provides classes for converting FBA-related files of different formats. |
| **sbrt.shell.text.chem** | Provides classes and interfaces for defining formats for chemistry related objects. |
| **sbrt.shell.text.fba** | Provides classes and interfaces for defining formats for objects relevant to Flux Balance Analysis. |

Classes in sbrt.shell.text.chem used by sbrt.shell.proc.fba

****IrrevRxnFormatV1****
  
          This class is used to format irreversible chemical reactions.

****IrrevRxnFormatV2****
  
          This class is used to format irreversible chemical reactions.

****RevRxnFormatV2****
  
          This class is used to format reversible chemical reactions.

Classes in sbrt.shell.text.chem used by sbrt.shell.text.chem

****CatalystFormat****
  
          This interface is used to represent the formats of catalysts
in chemical reactions.

****ChemSpeciesFormat****
  
          This interface is used to represent the formats of chemical
species.

****ChemSpeciesFormatV1****
  
          This class is a concrete implemenation of `ChemSpeciesFormat`.

****RxnNameFormat****
  
          This interface is used to represent formats for the
names of chemical reactions.

Classes in sbrt.shell.text.chem used by sbrt.shell.text.fba

****CatalystFormat****
  
          This interface is used to represent the formats of catalysts
in chemical reactions.

****ChemSpeciesFormat****
  
          This interface is used to represent the formats of chemical
species.

****RxnNameFormat****
  
          This interface is used to represent formats for the
names of chemical reactions.

****RxnNameFormatV1****
  
          This class is a concrete implementation of `RxnNameFormat`.

---


|  |  |  |
| --- | --- | --- |
| **Overview** \| **Package** \| Class \| **Use** \| **Tree** \| **Deprecated** \| **Index** \| **Help** | |  |
| PREV   NEXT | **FRAMES**    **NO FRAMES**     **All Classes** |  |


---
